# Brazilian Cerrado Soil Actinobacteria Ecology

**DOI:** 10.1155/2013/503805

**Published:** 2013-02-21

**Authors:** Monique Suela Silva, Alenir Naves Sales, Karina Teixeira Magalhães-Guedes, Disney Ribeiro Dias, Rosane Freitas Schwan

**Affiliations:** ^1^Department of Biology, Federal University of Lavras (UFLA), Campus Universitário, 37.200-000 Lavras, MG, Brazil; ^2^Department of Food Science, Federal University of Lavras (UFLA), Campus Universitário, 37.200-000 Lavras, MG, Brazil

## Abstract

A total of 2152 Actinobacteria strains were isolated from native Cerrado (Brazilian Savannah) soils located in Passos, Luminárias, and Arcos municipalities (Minas Gerais State, Brazil). The soils were characterised for chemical and microbiological analysis. The microbial analysis led to the identification of nine genera (*Streptomyces, Arthrobacter, Rhodococcus, Amycolatopsis, Microbacterium, Frankia, Leifsonia, Nakamurella,* and *Kitasatospora*) and 92 distinct species in both seasons studied (rainy and dry). The rainy season produced a high microbial population of all the aforementioned genera. The pH values of the soil samples from the Passos, Luminárias, and Arcos regions varied from 4.1 to 5.5. There were no significant differences in the concentrations of phosphorus, magnesium, and organic matter in the soils among the studied areas. Samples from the Arcos area contained large amounts of aluminium in the rainy season and both hydrogen and aluminium in the rainy and dry seasons. The Actinobacteria population seemed to be unaffected by the high levels of aluminium in the soil. Studies are being conducted to produce bioactive compounds from Actinobacteria fermentations on different substrates. The present data suggest that the number and diversity of Actinobacteria spp. in tropical soils represent a vast unexplored resource for the biotechnology of bioactives production.

## 1. Introduction

Actinobacteria are a distinct group of bacteria that are widely distributed in nature [1]. Currently [2], Actinobacteria comprise eight groups with 48 genera. Special attention has been given to this bacterial group in biotechnological applications, which are a natural result of their great metabolic diversity [3]. Actinobacteria are the most common source of antibiotics [4] and are a promising source of a wide range of enzymes, enzyme inhibitors, immunomodifiers, and vitamins [5]. In nature, Actinobacteria play an important role in the cycling of organic compounds and have been associated with soil organic matter production, including production of the black pigments called melanin, which are related to soil humic acid [6].

*Streptomyces* is the most common Actinobacteria genus found in soils and constitutes up to 90% of the isolates. However, new approaches for the isolation of soil Actinobacteria have revealed that other genera are also present in significant numbers. Many new species have been isolated, and most of the isolates are able to produce novel secondary metabolites [1]. Since the first antibiotic from an Actinobacterium, reported more than 50 years ago [7], more than 4000 new bioactive compounds have been discovered. The search for new species or strains of the Actinobacterium group is still of primary interest to the biotechnology field.

Actinobacteria taxonomy is extremely complex, and classification using only the traditional methods, which are based on morphological and physiological characteristics, has led to very heterogeneous suprageneric groups. Recently, three main approaches have been suggested to identify species of Actinobacteria: chemotaxonomy (differentiation of species by chemical composition), numerical taxonomy (differentiation of species by phenotypic similarity number), and molecular systematics (use of DNA study to the species differentiation). A combination of three techniques becomes more complete [1].

Brazilian Cerrado soils have an enormous biodiversity potential. Some of these soils have been described as habitats with high biological activity but have not been extensively explored for the search and discovery of novel Actinobacteria spp. In this study, isolation of Actinobacteria species from Brazilian Cerrado soils and comparisons of the Actinobacteria communities of the Cerrado soil with the physicochemical characteristics of these soils were performed. Some of the isolates that showed promise for use in biotechnology were identified and tested for the production of bioactives (e.g., enzymes and antibiotics).

## 2. Materials and Methods

### 2.1. Soil Sampling

**Figure 1:**
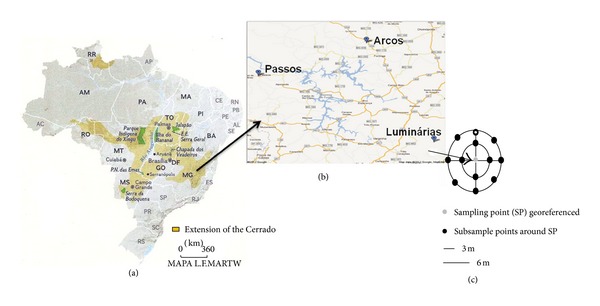
(a) Location of Cerrado soil in Minas Gerais, Brazil. (b) Cities where samples were collected. (c) Distribution of sampling point. Sampling point scheme: one composed soil sample (12 subsamples) was collected around each sampling point.

Thirty composite soil samples were collected during the months of January (rainy season) and August (dry season) from the Passos, Arcos, and Luminárias municipalities. These soils are highly conserved (native), and the locations are georeferenced in Table 1 and Figure 1. Each sample was obtained according to the procedure of Lima et al. [8]. Twelve subsamples of 75 to 100 g were collected from each point in two concentric circles with radii of 3 and 6 m from the centre and a depth of 0 to 20 cm using a flamed auger. The collected material from each point was mixed in a sterile bag and stored at 4°C until analysis.

### 2.2. Physicochemical Analysis of Soils

Approximately 200 g of each soil sample was subjected to physicochemical analysis using the procedure of the Embrapa [9]. The concentrations of potassium (K), phosphorus (P), aluminium (Al), magnesium (Mg), organic matter (OM), hydrogen and aluminium (Al + H) and exchangeable bases (SB) as well as the pH and soil texture were evaluated. The Sisvar 5.1 program of the SAS System 9.1 software (SAS Institute Inc., Cary, NC, USA) was used for statistical analysis of the differences between the means. Correlations between the Brazilian Cerrado soils' regions and the physicochemical soil variables were subjected to statistical analysis (principal component analysis or PCA) using the Unscrambler 9.7 software (CAMO, Oslo, Norway).

### 2.3. Bacterial Isolation and Culture Purification

Ten grams of soil from each composite sample was added to 90 mL of sterile peptone water (bacto peptone, 1 g/L) and homogenised by stirring at 130 rpm for 10 min (dilutions of 10^−1^ to 10^−8^). These sample dilutions were used for inoculations by spreading 100 *μ*L on the surface of Aaronson's medium according to Silva et al. [10] and humic acid vitamin medium according to Hayakawa and Nonomura [11]. The plates were incubated for 72 h to 120 h at 28°C.

From the plates containing 30 to 300 CFU, a number of colonies equal to the square root of the number of different quantified of each colonial morphotypes were isolated [13]. These morphotype strains were cultured for 72 to 120 h at 28°C in 500 *μ*L of nutrient broth supplemented with glycerol to a final concentration of 20%. The isolates were purified by successive restreak and were preserved by freezing at −20°C. The strains were reactivated on nutrient agar by incubating for 72 to 120 h at 28°C and were then characterised for bacterial colony morphology (i.e., size, shape, elevation, brightness, texture and colour) by making comparison between the colonies that were originally isolated from the culture media. The pure cultures were preserved under the conditions described above.

### 2.4. PCR Primer System for Selective Amplification of Actinobacteria

Pure cultures of the various colonial morphotypes were characterised by actinobacterial-specific primers according to Schäfer et al. [12] and described in Table 2. The 27f and 1492r universal bacterial primers were used as controls. The actinobacterial strains were subjected to molecular characterisation by REP-PCR as described below.

### 2.5. Molecular Characterisation Based on Repetitive Extragenic Palindromic-PCR (Rep-PCR)

Total genomic DNA was extracted as described by Pereira et al. [13]. The molecular characterisation of selected isolates was performed by polymerase chain reaction sequencing by REP-PCR as described by Gevers et al. [14]. Two microliters of DNA were added to 12.5 *μ*L of Taq PCR Master Mix (Qiagen, São Paulo, Brazil), 8 *μ*L H_2_O, 0.25 *μ*L bovine serum albumin (BSA), 0.25 *μ*L of formamide, and 2 *μ*L of primer GTG_5_ (5′-GTG GTG GTG GTG GTG-3′) [13]. PCR was performed under the following cycling conditions: 5 min initial denaturation at 94°C; 30 cycles of 95°C for 30 s, 45°C for 60 s and 60°C for 5 min; and a final elongation at 60°C for 16 min. The PCR products were separated by electrophoresis on a 2% agarose gel in 1x TAE buffer at 60 V for 4 h, stained with SYBR Green (Invitrogen, Foster City, CA, USA), and visualised under a transilluminator. The Rep-PCR profiles were subjected to cluster analysis using the Bionumerics 2.50 software (Applied Maths, Sint-Martens-Latem, Belgium).

### 2.6. PCR Amplification and DNA Sequencing of the 16S rRNA Gene

Representative isolates of each Rep-PCR profile were selected for amplification of the 16S rRNA gene as described by Pereira et al. [13]. DNA (2 *μ*L) was added to 30 *μ*L of Taq PCR Master Mix (Qiagen, São Paulo, Brazil), 26 *μ*L of H_2_O, 1 *μ*L of primer 27f (5′-AGAGTTTGATCCTGGCTCAG-3′), and 1 *μ*L of primer 1512r (5′-ACGGCTACCTTGTTACGACT-3′). The PCR reaction was performed as follows: initial denaturation at 95°C for 10 min; 25 cycles at 93°C for 1 min, 50°C for 1 min, and 72°C for 1 min 30 s; and a final elongation at 72°C for 5 min. The presence of PCR products was confirmed by electrophoresis on a 1% agarose gel in 1x TAE buffer at 70 V for 30 min, stained with SYBR Green (Invitrogen, Foster City, CA, USA), and visualised under a transilluminator. The sequencing of amplicons was performed at Macrogen Inc. (Seoul, South Korea), and the sequences were compared with the GenBank database using the BLAST algorithm (http://www.ncbi.nlm.nih.gov/BLAST/).

## 3. Results

### 3.1. Physicochemical Characteristics of the Brazilian Cerrado Soil Samples

The chemical and biochemical properties of the Cerrado soil from the Passos, Luminárias, and Arcos regions during the rainy and dry seasons are shown in Table 3. The pH values of the soils ranged from 4.7 to 5.5, 5.0 to 5.4, and 4.1 to 5.0 for the Passos, Luminárias, and Arcos regions, respectively. These soils had high acidity.

Differences in the soil textures from the Arcos, Passos, and Luminárias regions were observed. The three analysed areas revealed no significant differences in organic matter values. In general, the physical and chemical characteristics for all the soils analysed were similar.

**Figure 2:**
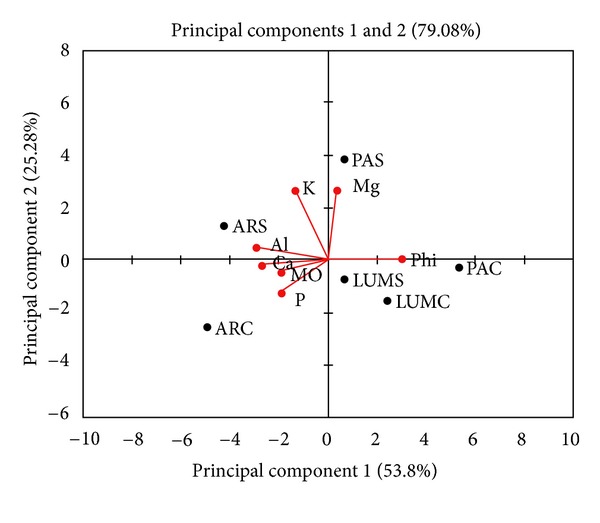
Principal component analysis (PCA) of chemical characteristics of the Brazilian Cerrado soil of Minas Gerais. Abbreviations: K = potassium; P = phosphorus; Al = aluminum, Ca = calcium, Mg = magnesium; PAC = Passos (rainy season); PAS = Passos (dry season); ARC = Arcos (rainy season); ARS = Arcos (dry season); LUMC = Luminárias (rainy season); LUMS = Luminárias (dry season).

A multivariate analysis using frequency values for the chemical characteristics of the Brazilian Cerrado soils was performed (Figure 2). Samples obtained from the Arcos region were significantly different from the Luminárias and Passos regional samples because of the high concentration of phosphorus during the rainy season and high concentrations of aluminium and potassium during the dry season in the Arcos region.

### 3.2. Microbial Isolation and Characterisation

Of the culture media tested (Aaronsons's medium and humic acid vitamin medium), all were able to recover colonies from all of the soil samples (Table 4).

Total bacterial counts were compared between the rainy and dry seasons. A statistically significant difference (*P* < 0.05) was observed for all the analysed areas (Table 4). The rainy season exhibited higher microbial counts (~9.1 log CFU/g) compared to the dry season (~7.9 log CFU/g). 

**Figure 3:**
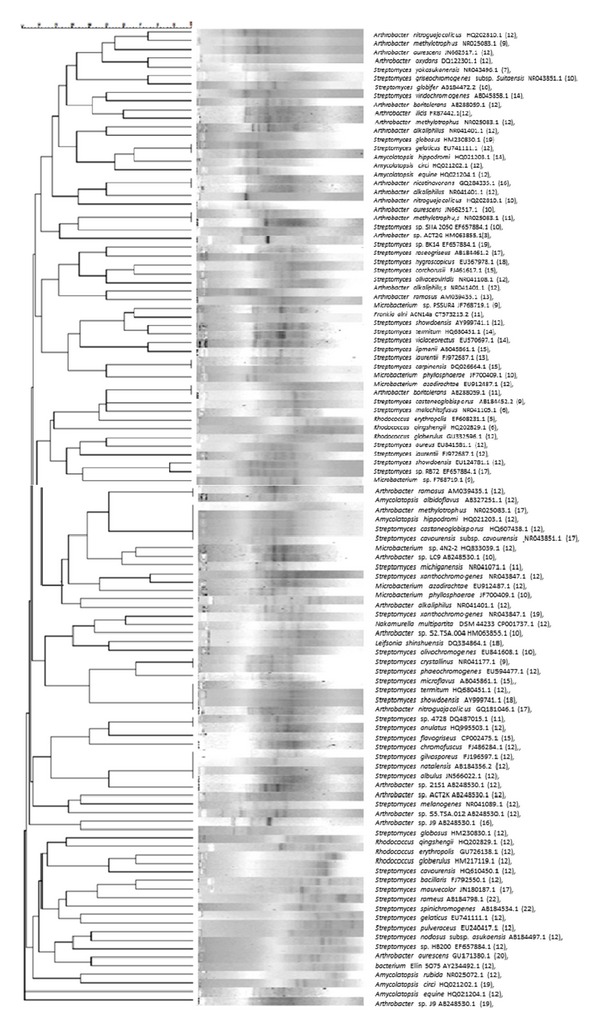
Similarity analysis between the bands' profiles (Rep-PCR) of the Actinobacteria isolates of the Brazilian Cerrado soils of three regions: Arcos, Passos, and Luminárias. ( ) OTUs quantification.

A total of 2152 isolates were characterised. The isolates were selected from the groups and subjected to group analysis corresponding to each region. The selected isolates from each region (188 isolates) were characterised by Rep-PCR, and 78 different band profiles were obtained (Figure 3).

### 3.3. Identification and Distribution of Isolates

The analysis of the 16S rRNA gene sequence amplification products led to the identification of nine Actinobacteria genera (*Streptomyces*, *Artrobacter*, *Rhodococcus*, *Amycolatopsis*, *Microbacterium*, *Frankia*, *Leifsonia*, *Nakamurella*, and *Kitasatospora*) and 92 distinct species within the genera (Figure 3 and Table 5).

The genera *Streptomyces*, *Artrobacter*, *Rhodococcus*, *Amycolatopsis*, and *Microbacterium* were found in all three regions analysed. *Leifsonia*, *Nakamurella*, and *Kitasatospora* were found only in the Arcos region, and the genus *Frankia* was not found in the Passos region (Table 5).

**Figure 4:**
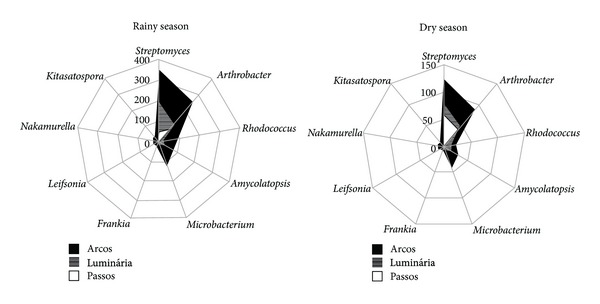
Actinobacteria genera abundance distribution in Cerrado soil in the rainy and dry seasons.

The distributions of Actinobacteria genera were different in the Cerrado soils during the rainy and dry seasons (776 isolates were from the dry season, and 1376 isolates were from the rainy season) (Table 5). The rainy season produced a higher microbial population for all described genera (Figure 4).

## 4. Discussion

The Cerrado biome has two distinct seasons: the dry season (May to September) and the rainy season (November to April). An important factor relevant to this study is that the soil samples were collected at the peak of each season; thus, the distinction between the samples was maximised because water was either limited or abundant [15]. The samples were collected in January (high rainfall, 1000–2100 mm) and August (low rainfall, 20–200 mm) of 2010. An important caveat is the heterogeneity of the distribution of microorganisms in the soil because microbial growth is usually observed in patches rather than homogeneously [16–18]. We minimised this potential bias by collecting samples from different spots in each area studied and mixing the individual samples to obtain a composite sample.

Microorganisms are the key drivers of biogeochemical processes in the soil. Thus, it is important to evaluate the physicochemical properties of the soil and how these properties could be related to microbial profiles in different soils [19]. These changes in the soil affect the native microbial populations. Seasonal variations in the moisture and pH of the soil can lead to changes in the distribution patterns of the microbial species. For example, bacteria prefer neutral to alkaline conditions, whereas yeasts and filamentous fungi prefer acidic conditions. Some microbial species also have preferences for soils with high or low moisture contents [20]. These Cerrado soils had high acidity, consistent with the values found in Cerrado soils that have been reported by studies of others [15, 21]. The Cerrado soils are commonly acids. This may be due to the vegetative and microbial population present [15, 21]. Despite having low pH, large amounts of aluminium and iron, and low nutrient content, these soils are extensively used in agriculture. One of the few studies dealing with the *Microbiota* of these soils reported high numbers of Actinobacteria [21].

The pH values of the Cerrado soils were similar; however, moisture influenced the total microbial population counts. Samples collected in the rainy season are contained in a higher microbial population (~9.1 log CFU/g) than those collected in the dry season (~7.9 log CFU/g) (Table 4).

The Arcos regional soil contained large amounts of aluminium (~2.4 mg/dm^3^) during the rainy season and large amounts of hydrogen and aluminium during the rainy and dry seasons (~15 Cmol/dm^3^) (Table 3). High quantities of soluble aluminium in the soil can cause toxicity in plants, as aluminium competes with other elements (e.g., essential nutrients) for the same chemical sites and promotes soil impoverishment [22]. However, in this study, the *Microbiota* was not affected by high aluminium levels in the soil based on the similarity of this population to those at the other sampled sites. The three analysed areas (Luminárias, Arcos, and Passos) displayed no significant differences in organic matter contents, which may be due to the similarity in the vegetation and riverbank forest profiles of the three areas [15].

Brazilian Cerrado soils cover a vast area, representing up to 25% of the country [1]. Despite having a low pH (~5.0), large amounts of aluminium and iron, and a low nutrient content, these soils are extensively used in agriculture. One of the few studies examining the microbial population of these soils reported on high numbers of Actinobacteria [21]. Huddleston and collaborators [23] found a culturable streptomycete population of approximately 8.0 log CFU/g in soil. In the previous study, the number of Actinobacteria isolated from those soils was on the same order of magnitude (~8.8 log CFU/g of soil) as the number isolated from soils of the Brazilian Cerrado regions of the Minas Gerais State (Luminárias, Passos, and Arcos). These findings suggest that the Cerrado soils represent a large, unexplored environment for the potential isolation of Actinobacteria. Morphological and physiological data led to either a partial or complete identification of 2152 isolates from the Cerrado soil. Dendrograms of the identified strains indicated that they were phenotypically diverse from the Actinobacteria species already described with the majority clustering in a separate and isolated group. Tropical soils present a myriad of microhabitats scarcely explored microbiologically. According to Zucchi et al. [1], of the 16,013 fungal species described as new to science over a ten-year period (from 1981 to 1990), 49% of the species were from tropical countries. This observation may be extended to other microbial groups, including Actinobacteria, for which there are no statistics available concerning Brazilian tropical soils.

Actinobacteria are Gram-positive, morphologically and physiologically very diverse bacteria with a high GC content in their DNA, and they are one of the main phyla within the domain Bacteria. The class Actinobacteria contains six orders—Acidimicrobiales, Rubrobacterales, Coriobacterales, Bifidobacteriales, Actinomycetales, and Nitriliruptorales. Actinobacteria are dominant colonizers in soils. Many species produce extracellular enzymes for degradation of macromolecules such as lignin, cellulose, chitin, and, in part, starch. Therefore, Actinobacteria often occur in materials where organic materials are degraded [12]. In particular, investigations in the indoor environment demonstrated their presence in water-damaged building materials and soils beside fungi [12]. This may explain the high presence of Actinobacteria in these soils of Brazilian Cerrado. In nature, Actinobacteria play an important role in the cycling of organic compounds and have also been associated with soil organic matter production, owing to their black pigments called melanins, which are related, in some respects, to soil humic acid [12, 21].

In the present work, the soils studied were characterised as being especially rich in the *Streptomyces* genus, as are other soils throughout the world. The *Streptomyces* genus has been the focus of research because of the commercial applicability of substances produced as well as the systematics of this group, which have been modified with advances in molecular biology [24]. Among the species isolated, *S. cavourensis* is a producer of the antibiotic chromomycin [25] and *S. michiganensis* is involved in the synthesis of anthelmintic and antiprotozoal substances [26].

Members of the *Arthrobacter* genus are widely distributed in ecosystems and can be isolated from diverse environments, such as air, water (fresh and salt), soil, oil, airborne infections, tobacco leaves, human skin, and activated sludge. *Arthrobacter* spp. exhibit great metabolic versatility and are able to degrade pollutants and xenobiotics, such as heavy metals (As, Cd, Cr, Cu, Hg, Ni, Pb, Se, V, and Zn) [27]. One of the species identified in this work, *A. ramosus*, is involved in the synthesis of protease, an enzyme important for the food, pharmaceutical, leather, and detergent industries. *A. ramosus* is also highly resistant to a variety of heavy metals and may be useful for bioremediation processes [27].

Species of the *Microbacterium* genus can be isolated from air, soil, water, fungi, plants, and humans. Many *Microbacterium* spp. play a significant role in human health, industry, agriculture, environment, bioengineering, and biotechnology and have applicability in the production of exopolysaccharide, degradation of oil, degradation of xylan, metal tolerance, production of biosurfactants, degradation of dimethylsulphide, degradation of lactone, and as a growth promoter in plants [28]. *Microbacterium phyllosphaerae* was found in all three regions examined in this study. In combination with *Burkholderia* sp. and *Candida tropicallis*, *M. phyllosphaerae* performs the processes responsible for the biodegradation of chlorophenols, which are commonly used in the chemical industry as intermediates in the synthesis of insecticide, fungicide, and herbicide and cause serious environmental damage [28].

The strains identified in this study have been previously characterised as important for biotechnology applications. Studies are currently being conducted to produce bioactive compounds from Actinobacteria fermentations on different substrates. The present data suggest that the number and diversity of Actinobacteria in tropical soils represent a vast unexplored resource for the biotechnology of bioactives production.

## 5. Future Perspectives

We commented on the introduction on the global development of soil microbiology and the renaissance taking place in natural product research. Furthermore, we reiterated our belief that natural product search and discovery with soil Actinobacteria shows exceptional promise. Our optimism is based on the spectacular technological armamentarium that is now available as well as the relatively complete but slowly developing understanding of soil biology. This optimism is also encouraged by the wide range of natural products that may exist with a diversity of applications (e.g., enzymes [5], antibiotics [4], fertilizer, pesticide [16], etc.). However, in this study, the focus was on the microbial diversity of Actinobacteria. Soil actinobacterial research and discovery is an important component of natural product research but the development of discoveries to yield products must also be addressed. Although there are encouraging signs that the newer biotechnology companies are focusing on soil organisms, medical necessity as much as business opportunity should ultimately be the driver behind investment in natural product drugs.

## Figures and Tables

**Table 1:** Location and description of the Brazilian Cerrado soil collection sites.

Site name	Location
Region of Passos	
Point 1	20°49′57.7′′S; 046°30′29.3′′W
Point 2	20°49′56.8′′S; 046°30′30.1′′W
Point 3	20°49′48.0′′S; 046°30′54.9′′W
Point 4	20°49′47.1′′S; 046°30′54.5′′W
Point 5	20°49′47.8′′S; 046°30′51.5′′W
Region of Luminárias	
Point 6	21°37′51.0′′S; 044°58′22.7′′W
Point 7	21°37′50.6′′S; 044°58′22.7′′W
Point 8	21°37′51.5′′S; 044°59′11.0′′W
Point 9	21°37′55.3′′S; 044°59′29.3′′W
Point 10	21°37′54.6′′S; 044°59′54.0′′W
Region of Arcos	
Point 11	20°16′27.7′′S; 045°29′14.6′′W
Point 12	20°14′47.9′′S; 045°25′35.9′′W
Point 13	20°14′51′′S; 045°31′40.8′′W
Point 14	20°14′48.6′′S; 045°31′33.4′′W
Point 15	20°14′58.0′′S; 045°31′54.0′′W

**Table 2:** Actinobacterial-specific primer used in bacterial communities in the Brazilian Cerradosoils, according to Schäfer et al. [12].

Primer	Sequence (5′–3′)	Primers	Amplified fragment (bp)	PCR conditions
27f	GAG TTT GAT CMT GGC TCA G	Bacterial universal primer	~1500	Denatured for 5 min at 95°C. 30 cycles: denaturing at 92°C for 60 s, annealing at 55°C for 60 s, and extension at 72°C for 60 s; final extension for 10 min at 72°C
1492r	ACG GYT ACC TTG TTA CGA CTT			
Com2xf	AAA CTC AAA GGA ATT GAC GG	Actinobacterial-specific primer	~270	
Ac1186r	CTT CCT CCG AGT TGA CCC			
SC-Act-235aS20	CGC GGC CTA TCA GCT TGT TG	Actinobacterial-specific primer	~640	
SC-Act-878aA19	CCG TAC TCC CCA GGC GGG G			

**Table 3:** Chemical and physical characteristics of the Brazilian Cerrado soil samples.

Sample and Season			pH	P (mg/dm^3^)	K (mg/dm^3^)	Mg (mg/dm^3^)	Al (mg/dm^3^)	H + Al (Cmol/dm^3^)	OM (dag/Kg)	SB (mg/dm^3^)	Texture
PA	Rainy	Point 1	5.3 ± 0.1^a^	1.5 ± 0.1^a^	25 ± 1^a^	0.1 ± 0.0^a^	0.6 ± 0.1^a^	3.6 ± 0.1^a^	1.4 ± 0.1^a^	0.3 ± 0.1^a^	Sandy loam
		Point 2	5.4 ± 0.1^a^	1.5 ± 0.1^a^	56 ± 2^a^	0.1 ± 0.0^a^	0.6 ± 0.1^a^	4.5 ± 0.1^a^	2.0 ± 0.1^a^	0.4 ± 0.1^a^	Medium loam
		Point 3	5.5 ± 0.1^a^	1.2 ± 0.1^a^	33 ± 1^a^	0.2 ± 0.0^a^	0.4 ± 0.1^a^	2.6 ± 0.1^a^	1.1 ± 0.1^a^	0.3 ± 0.1^a^	Medium loam
		Point 4	5.5 ± 0.1^a^	1.0 ± 0.1^a^	70 ± 1^b^	0.1 ± 0.0^a^	0.5 ± 0.1^a^	3.6 ± 0.1^a^	1.5 ± 0.1^a^	0.5 ± 0.1^a^	Medium loam
		Point 5	5.4 ± 0.1^a^	0.7 ± 0.1^a^	9 ± 1^b^	0.1 ± 0.0^a^	0.1 ± 0.1^a^	1.7 ± 0.1^a^	0.4 ± 0.1^b^	0.2 ± 0.1^a^	Medium loam

LU	Rainy	Point 6	5.4 ± 0.1^a^	1.2 ± 0.1^a^	28 ± 1^a^	0.2 ± 0.0^a^	0.5 ± 0.1^a^	7.9 ± 0.1^a^	3.4 ± 0.1^a^	0.3 ± 0.1^a^	Clay loam
		Point 7	5.0 ± 0.1^a^	1.5 ± 0.1^a^	20 ± 1^a^	0.1 ± 0.0^a^	0.5 ± 0.1^a^	7.9 ± 0.1^a^	2.6 ± 0.1^a^	0.3 ± 0.1^a^	Clay loam
		Point 8	5.1 ± 0.1^a^	1.2 ± 0.1^a^	11 ± 1^b^	0.2 ± 0.0^a^	0.3 ± 0.1^a^	2.6 ± 0.1^a^	1.1 ± 0.1^a^	0.2 ± 0.1^a^	Sandy loam
		Point 9	5.2 ± 0.1^a^	2.0 ± 0.1^a^	20 ± 1^a^	0.1 ± 0.0^a^	0.9 ± 0.2^a^	7.0 ± 0.1^a^	2.4 ± 0.1^a^	0.3 ± 0.1^a^	Medium loam
		Point 10	5.1 ± 0.1^a^	1.5 ± 0.1^a^	34 ± 1^a^	0.1 ± 0.0^a^	0.8 ± 0.1^a^	8.8 ± 0.3^b^	2.7 ± 0.1^a^	0.3 ± 0.1^a^	Clay loam

AC	Rainy	Point 11	5.0 ± 0.1^a^	1.2 ± 0.1^a^	48 ± 1^a^	0.1 ± 0.0^a^	0.6 ± 0.1^a^	4.0 ± 0.1^a^	1.6 ± 0.1^a^	0.7 ± 0.1^a^	Clay loam
		Point 12	4.6 ± 0.1^a^	0.7 ± 0.1^a^	39 ± 1^a^	0.1 ± 0.0^a^	1.0 ± 0.1^a^	6.3 ± 0.1^a^	2.0 ± 0.1^a^	0.3 ± 0.1^a^	Clay loam
		Point 13	4.1 ± 0.1^a^	1.8 ± 0.1^a^	27 ± 1^a^	0.3 ± 0.0^a^	2.1 ± 0.1^b^	15.3 ± 1^b^	3.4 ± 0.1^a^	0.3 ± 0.1^a^	Clay loam
		Point 14	4.1 ± 0.1^a^	1.8 ± 0.1^a^	33 ± 1^a^	0.1 ± 0.0^a^	2.4 ± 0.1^b^	17.1 ± 2^b^	4.0 ± 0.1^a^	0.3 ± 0.1^a^	Clay loam
		Point 15	5.0 ± 0.1^a^	1.8 ± 0.1^a^	69 ± 2^b^	0.1 ± 0.0^a^	1.8 ± 0.1^b^	12.3 ± 1^b^	2.7 ± 0.1^a^	0.4 ± 0.1^a^	Clay loam

PA	Dry	Point 1	4.7 ± 0.1^a^	1.7 ± 0.1^a^	113.8 ± 1^b^	0.1 ± 0.0^a^	0.2 ± 0.1^a^	13.7 ± 0.1^b^	3.9 ± 0.1^a^	0.5 ± 0.1^a^	Sandy loam
		Point 2	5.1 ± 0.1^a^	1.7 ± 0.1^a^	88.9 ± 1^b^	0.1 ± 0.0^a^	0.4 ± 0.1^a^	5.6 ± 0.1^a^	2.4 ± 0.1^a^	0.7 ± 0.1^a^	Medium loam
		Point 3	5.1 ± 0.1^a^	1.4 ± 0.1^a^	137.28 ± 1^b^	0.1 ± 0.0^a^	0.4 ± 0.1^a^	4.5 ± 0.1^a^	2.2 ± 0.1^a^	0.8 ± 0.1^a^	Medium loam
		Point 4	5.1 ± 0.1^a^	1.7 ± 0.1^a^	117 ± 1^b^	0.1 ± 0.0^a^	0.5 ± 0.1^a^	5.0 ± 0.1^a^	1.9 ± 0.1^a^	0.9 ± 0.1^a^	Medium loam
		Point 5	5.2 ± 0.1^a^	1.4 ± 0.1^a^	54 ± 1^b^	0.1 ± 0.0^a^	0.2 ± 0.1^a^	4.5 ± 0.1^a^	1.7 ± 0.1^a^	0.4 ± 0.1^a^	Medium loam

LU	Dry	Point 6	5.1 ± 0.1^a^	2.5 ± 0.1^a^	37.4 ± 1^a^	0.1 ± 0.0^a^	0.6 ± 0.1^a^	6.3 ± 0.1^a^	2.2 ± 0.1^a^	0.1 ± 0.1^a^	Clay loam
		Point 7	5.1 ± 0.1^a^	0.9 ± 0.1^a^	37.4 ± 1^a^	0.1 ± 0.0^a^	1.5 ± 0.1^b^	7.0 ± 0.1^a^	2.8 ± 0.1^a^	0.2 ± 0.1^a^	Clay loam
		Point 8	5.2 ± 0.1^a^	0.9 ± 0.1^a^	39 ± 1^a^	0.1 ± 0.0^a^	0.7 ± 0.1^a^	7.8 ± 0.1^a^	2.8 ± 0.1^a^	0.2 ± 0.1^a^	Sandy loam
		Point 9	5 ± 0.1^a^	1.2 ± 0.1^a^	46 ± 1^a^	0.1 ± 0.0^a^	1.5 ± 0.1^b^	10.9 ± 0.1^b^	3.0 ± 0.1^a^	0.3 ± 0.1^a^	Medium loam
		Point 10	5 ± 0.1^a^	1.2 ± 0.1^a^	67 ± 1^b^	0.1 ± 0.0^a^	1.4 ± 0.1^b^	9.88 ± 0.1^b^	3.1 ± 0.1^a^	0.3 ± 0.1^a^	Clay loam

AC	Dry	Point 11	4.7 ± 0.1^a^	2.0 ± 0.1^a^	149.7 ± 1^b^	0.1 ± 0.0^a^	0.4 ± 0.1^a^	8.7 ± 0.1^a^	2.4 ± 0.1^a^	1.3 ± 0.1^a^	Clay loam
		Point 12	4.8 ± 0.1^a^	1.4 ± 0.1^a^	48.3 ± 1^a^	0.6 ± 0.0^a^	0.1 ± 0.1^a^	7.0 ± 0.1^a^	1.8 ± 0.1^a^	0.2 ± 0.1^a^	Clay loam
		Point 13	4.3 ± 0.1^a^	1.4 ± 0.1^a^	54.6 ± 1^a^	0.1 ± 0.0^a^	0.1 ± 0.1^a^	15.3 ± 1^b^	2.8 ± 0.1^a^	0.3 ± 0.1^a^	Clay loam
		Point 14	4.2 ± 0.1^a^	1.7 ± 0.1^a^	39.0 ± 1^a^	0.1 ± 0.0^a^	0.1 ± 0.1^a^	17.1 ± 1^b^	3.0 ± 0.1^a^	0.2 ± 0.1^a^	Clay loam
		Point 15	4.8 ± 0.1^a^	1.2 ± 0.1^a^	84.2 ± 2^b^	0.1 ± 0.0^a^	0.1 ± 0.1^a^	10.9 ± 1^b^	1.9 ± 0.1^a^	0.4 ± 0.1^a^	Clay loam

Data are average values of duplicate ± standard deviation. Different letters indicate significant differences (*P* < 0.05). Soil classification in sandy (content clay <15), Medium (content clay between 15 and 35), and clay (content clay ≥35). Abbreviations: PA: Passos; LU: Luminárias; AC: Arcos. K: potassium; P: phosphorus; Al: aluminum; Ca: calcium; Mg: magnesium; H + Al: hydrogen + aluminum; OM: organic matter; SB: (exchangeable bases) the sum of Ca, Mg, Na, and K.

**Table 4:** Actinobacteria count of the population in log CFU/g of soil in differents medium during the rainy and dry season.

	Region	
	Aaronsons's medium	Humic acid vitamin medium
	Dry season	

Arcos	7.9 ± 0.1^a^	6.6 ± 0.1^b^
Luminárias	7.8 ± 0.1^a^	6.8 ± 0.1^b^
Passos	7.8 ± 0.1^a^	6.7 ± 0.1^b^

	Rainy season	

Arcos	9.1 ± 0.2^c^	7.2 ± 0.1^d^
Luminárias	8.9 ± 0.2^c^	7.1 ± 0.1^d^
Passos	8.8 ± 0.1^c^	7.2 ± 0.2^d^

Data are mean values of duplicate ± standard deviation.

Different letters indicate significant differences (*P* < 0.05).

**Table 5:** Actinobacteria diversity in different Cerrado regions of Minas Gerais, Brazil. OTUs and abundance quantification.

Region	Season	Total abundance	Actinobacteria
Passos	Rainy	153	*Arthrobacter nitroguajacolicus* HQ202810.1 (12), *Arthrobacter methylotrophus *NR025083.1 (9), *Arthrobacter aurescens *JN662517.1 (12), *Arthrobacter oxydans *DQ122301.1 (12), *Streptomyces yokosukanensis *NR043496.1 (7), *Streptomyces griseochromogenes *subsp*. suitaensis *NR043851.1 (10), *Streptomyces globifer* AB184472.2 (10), *Streptomyces viridochromogenes *AB045858.1 (14), *Arthrobacter boritolerans *AB288059.1 (12), *Arthrobacter ilicis* FR87442.1 (12), *Arthrobacter methylotrophus *NR025083.1 (12), *Arthrobacter alkaliphilus *NR041401.1 (12), *Streptomyces globosus *HM230830.1 (19)
	Dry	105	*Arthrobacter nitroguajacolicus* HQ202810.1 (10), *Arthrobacter methylotrophus *NR025083.1 (11), *Arthrobacter aurescens * JN662517.1 (11), *Arthrobacter oxydans* DQ122301.1 (9), *Streptomyces yokosukanensis *NR043496.1 (12), *Streptomyces griseochromogenes *subsp*. suitaensis* NR043851.1 (10), *Streptomyces globifer* AB184472.2 (12), *Streptomyces viridochromogenes* AB045858.1 (5), *Arthrobacter boritolerans* AB288059.1 (4), *Arthrobacter ilicis *FR87442.1 (4), *Arthrobacter methylotrophus* NR025083.1 (1), *Arthrobacter alkaliphilus* NR041401.1 (9),* Streptomyces globosus* HM230830.1 (7)

Luminárias	Rainy	414	*Streptomyces gelaticus* EU741111.1 (12), *Amycolatopsis hippodromi *HQ021203.1 (14), *Amycolatopsis circi *HQ021202.1 (12), *Amycolatopsis equine *HQ021204.1 (12), *Arthrobacter nicotinovorans* GQ284335.1 (16), *Arthrobacter alkaliphilus *NR041401.1 (12), *Arthrobacter nitroguajacolicus* HQ202810.1 (10), *Arthrobacter aurescens* JN662517.1 (10), *Arthrobacter methylotrophus* NR025083.1 (11), *Streptomyces *sp. SIIA 2050 EF657884.1 (10), *Arthrobacter *sp. ACT2G HM063855.1 (3), *Streptomyces *sp. BK14 EF657884.1 (19), *Streptomyces roseogriseus* AB184461.2 (17), *Streptomyces hygroscopicus *EU367978.1 (18), *Streptomyces corchorusii* FJ461617.1 (15), *Streptomyces olivaceoviridis* NR041108.1 (12), *Arthrobacter alkaliphilus* NR041401.1 (12), *Arthrobacter ramosus* AM039435.1 (13), *Microbacterium *sp. PSSUR4 JF768719.1 (9), *Frankia alni *ACN14a CT573213.2 (11), *Streptomyces showdoensis* AY999741.1 (12), *Streptomyces termitum* HQ680451.1 (14), *Streptomyces violaceorectus *EU570697.1 (14), *Streptomyces lipmanii* AB045861.1 (15), *Streptomyces laurentii* FJ972687.1 (13), *Streptomyces carpinensis* DQ026664.1 (15), *Microbacterium phyllosphaerae* JF700409.1 (10), *Microbacterium azadirachtae *EU912487.1 (12), *Arthrobacter boritolerans* AB288059.1 (11), *Streptomyces castaneoglobisporus* AB184452.2 (9), *Streptomyces malachitofusus* NR041105.1 (6), *Rhodococcus erythropolis* EF608231.1 (5), *Rhodococcus qingshengii* HQ202829.1 (6), *Rhodococcus globerulus* GU332596.1 (12), *Streptomyces aureus *EU841581.1 (12),
	Dry	245	*Streptomyces gelaticus* EU741111.1 (10), *Amycolatopsis hippodromi* HQ021203.1 (9), *Amycolatopsis circi* HQ021202.1 (7), *Amycolatopsis equine* HQ021204.1 (5)*, Arthrobacter nicotinovorans* GQ284335.1 (4)*, Arthrobacter alkaliphilus* NR041401.1 (10)*, Arthrobacter nitroguajacolicus* HQ202810.1 (11), *Arthrobacter aurescens* JN662517.1 (9)*, Arthrobacter methylotrophus* NR025083.1 (12)*, Streptomyces *sp. SIIA 2050 EF657884.1 (9), *Arthrobacter *sp. ACT2G HM063855.1 (1), *Streptomyces *sp. BK14 EF657884.1 (7), *Streptomyces roseogriseus* AB184461.2 (8)*, Streptomyces hygroscopicus* EU367978.1 (6), *Streptomyces corchorusii* FJ461617.1 (5)*, Streptomyces olivaceoviridis* NR041108.1 (8)*, Arthrobacter alkaliphilus* NR041401.1 (4)*, Arthrobacter ramosus* AM039435.1 (12), *Microbacterium *sp. PSSUR4 JF768719.1 (7), *Frankia alni *ACN14a CT573213.2 (9), *Streptomyces showdoensis* AY999741.1 (10), *Streptomyces termitum* HQ680451.1 (10)*, Streptomyces violaceorectus* EU570697.1 (11)*, Streptomyces lipmanii* AB045861.1 (9)*, Streptomyces laurentii* FJ972687.1 (12)*, Streptomyces carpinensis* DQ026664.1 (12), *Microbacterium phyllosphaerae* JF700409.1 (4), *Microbacterium azadirachtae *EU912487.1 (7)*, Arthrobacter boritolerans* AB288059.1 (2), *Streptomyces castaneoglobisporus* AB184452.2 (2) *,Streptomyces malachitofusus* NR041105.1 (2)*, Rhodococcus erythropolis* EF608231.1 (2)*, Rhodococcus qingshengii* HQ202829.1 (2)*, Rhodococcus globerulus* GU332596.1 (3)*, Streptomyces aureus* EU841581.1 (4),

Arcos	Rainy	865	*Arthrobacter ramosus* AM039435.1 (12)*, Amycolatopsis albidoflavus *AB327251.1 (12)*, Arthrobacter methylotrophus *NR025083.1 (17)*, Amycolatopsis hippodromi* HQ021203.1 (12), S*treptomyces castaneoglobisporus* HQ607438.1 (12), *Streptomyces cavourensis * subsp. *cavourensis* NR043851.1 (17), *Microbacterium *sp. 4N2-2 HQ833039.1 (12)*, Arthrobacter *sp. LC9 AB248530.1 (10)*, Streptomyces michiganensis *NR041071.1 (11)*, Streptomyces xanthochromogenes* NR043847.1 (12), *Microbacterium azadirachtae *EU912487.1 (12), *Microbacterium phyllosphaerae *JF700409.1 (10)*, Arthrobacter alkaliphilus *NR041401.1 (12), * Streptomyces xanthochromogenes *NR043847.1 (19)*, Nakamurella multipartita *DSM 44233 CP001737.1 (12)*, Arthrobacter *sp. S2.TSA.004 HM063855.1 (10)*, Leifsonia shinshuensis *DQ334864.1 (18)*, Streptomyces olivochromogenes *EU841608.1 (10), *Streptomyces crystallinus* NR041177.1 (9)*, Streptomyces phaeochromogenes *EU594477.1 (12)*, Streptomyces microflavus *AB045861.1 (15), *Streptomyces termitum* HQ680451.1 (12)*, Streptomyces showdoensis *AY999741.1 (18)*, Arthrobacter nitroguajacolicus* GQ181046.1 (17)*, Streptomyces *sp. 4728 DQ487015.1 (11), *Streptomyces anulatus *HQ995503.1 (12), *Streptomyces flavogriseus *CP002475.1 (15), *Streptomyces chromofuscus* FJ486284.1 (12), *Streptomyces gilvosporeus* FJ196597.1 (12), *Streptomyces natalensis* AB184356.2 (12), *Streptomyces albulus* JN566022.1 (12), *Arthrobacter *sp. 21S1 AB248530.1 (12), *Arthrobacter *sp. ACT2K AB248530.1 (12)*, Streptomyces melanogenes* NR041089.1 (12), *Arthrobacter *sp. S5.TSA.012 AB248530.1 (12), *Arthrobacter *sp. J9 AB248530.1 (16), *Streptomyces globosus* HM230830.1 (12), *Rhodococcus qingshengii *HQ202829.1 (12), *Rhodococcus erythropolis* GU726138.1 (12), *Rhodococcus globerulus *HM217119.1 (12), *Streptomyces cavourensis* HQ610450.1 (12)*, Streptomyces bacillaris *FJ792550.1 (12), *Streptomyces mauvecolor* JN180187.1 (17)*, Streptomyces rameus *AB184798.1 (22)*, Streptomyces spinichromogenes *AB184534.1 (22)*, Streptomyces gelaticus *EU741111.1 (12)*, Streptomyces pulveraceus *EU240417.1 (12)*, Streptomyces nodosus *subsp*. asukaensis* AB184497.1 (12), *Streptomyces *sp. HB200 EF657884.1 (12), *Arthrobacter aurescens* GU171380.1 (20)*, bacterium *Ellin 5075 AY234492.1 (12), *Amycolatopsis rubida *NR025072.1 (12)*, Amycolatopsis circi *HQ021202.1 (19)*, Amycolatopsis equine *HQ021204.1 (12)*, Streptomyces laurentii *FJ972687.1 (12)*, Streptomyces showdoensis *EU124781.1 (12)*, Streptomyces *sp. RB72 EF657884.1 (17), *Streptomyces xanthocidicus* NR043370.1 (12)*, Streptomyces tsukiyonensis* AB184594.1 (14), *Kitasatospora kifunensis *AJ781341.1 (12)*, Streptomyces recifensis* HM062992.1 (19)
	Dry	370	*Arthrobacter ramosus* AM039435.1 (10)*, Amycolatopsis albidoflavus* AB327251.1 (10)*, Arthrobacter methylotrophus * NR025083.1 (12)*, Amycolatopsis hippodromi* HQ021203.1 (12)*, Streptomyces castaneoglobisporus* HQ607438.1 (9), *Streptomyces cavourensis* subsp. *cavourensis* NR043851.1 (8)*, Microbacterium *sp. 4N2-2 HQ833039.1 (12)*, Arthrobacter *sp. LC9 AB248530.1 (12)*, Streptomyces michiganensis* NR041071.1 (12)*, Streptomyces xanthochromogenes* NR043847.1 (12), *Microbacterium azadirachtae* EU912487.1 (12)*, Microbacterium phyllosphaerae * JF700409.1 (3)*, Arthrobacter alkaliphilus* NR041401.1 (4)*, Streptomyces xanthochromogenes* NR043847.1 (3)*, Nakamurella multipartita *DSM 44233 CP001737.1 (2)*, Arthrobacter *sp. S2.TSA.004 HM063855.1 (12)*, Leifsonia shinshuensis* DQ334864.1 (10)*, Streptomyces olivochromogenes* EU841608.1 (12), *Streptomyces crystallinus* NR041177.1 (12), *Streptomyces phaeochromogenes* EU594477.1 (12)*, Streptomyces microflavus* AB045861.1 (12), *Streptomyces termitum* HQ680451.1 (12)*, Streptomyces showdoensis* AY999741.1 (2), *Arthrobacter nitroguajacolicus* HQ202810.1 (2)*, Streptomyces *sp. 4728 DQ487015.1 (12), *Streptomyces anulatus* HQ995503.1 (3), *Streptomyces flavogriseus* CP002475.1 (19), *Streptomyces chromofuscus * FJ486284.1 (13), *Streptomyces gilvosporeus* FJ196597.1 (14), *Streptomyces natalensis* AB184356.2 (7), *Streptomyces albulus* JN566022.1 (5), *Arthrobacter *sp. 21S1 AB248530.1 (3), *Arthrobacter *sp. ACT2K AB248530.1 (9), *Streptomyces melanogenes* NR041089.1 (12)*, Arthrobacter *sp. S5.TSA.012 AB248530.1 (12), *Arthrobacter *sp. J9 AB248530.1 (12), *Streptomyces globosus* HM230830.1 (12)*, Rhodococcus qingshengii* HQ202829.1 (7), *Rhodococcus erythropolis* GU726138.1 (2), *Rhodococcus globerulus* HM217119.1 (1)*, Streptomyces cavourensis* HQ610450.1 (2)*, Streptomyces bacillaris* FJ792550.1 (1), *Streptomyces mauvecolor* JN180187.1 (1), *Streptomyces rameus* AB184798.1 (1)*, Streptomyces spinichromogenes* AB184534.1 (2)*, Streptomyces gelaticus* EU741111.1 (2)*, Streptomyces pulveraceus* EU240417.1 (2)*, Streptomyces nodosus *subsp*. asukaensis* AB184497.1 (2), *Streptomyces *sp. HB200 EF657884.1 (2), *Arthrobacter aurescens* GU171380.1 (12)*, bacterium *Ellin 5075 AY234492.1 (1), *Amycolatopsis rubida* NR025072.1 (3)*, Amycolatopsis circi* HQ021202.1 (4)*, Amycolatopsis equine* HQ021204.1 (3)*, Streptomyces laurentii* FJ972687.1 (3)*, Streptomyces showdoensis* EU124781.1 (5), *Streptomyces *sp. RB72 EF657884.1 (6), *Streptomyces xanthocidicus* NR043370.1 (4), *Streptomyces tsukiyonensis* AB184594.1 (3), *Kitasatospora kifunensis* AJ781341.1 (2)*, Streptomyces recifensis* HM062992.1 (1)

( ) Distribution of isolates OTUs. Access Number—99% of similarity.
